# The relationship between the duration and the retraction and atrophy grades in traumatic isolated full-thickness supraspinatus tears in young patients

**DOI:** 10.1186/s12891-024-07659-9

**Published:** 2024-07-12

**Authors:** Gokhan Ilyas, Fikri Burak Ipci, Oguzhan Gokalp, Ercument Egeli

**Affiliations:** 1 Faculty of Medicine, Department of Orthopaedics and Traumatology, Usak University, Usak, Turkey; 2 Orthopaedics and Traumatology Clinic, Usak Esme State Hospital, Usak, Turkey

**Keywords:** Rotator cuff injuries, Shoulder joint, MRI scans, Muscle atrophy, Tendinopathy

## Abstract

**Background:**

The study aimed to determine the grade of retraction and atrophy according to the time elapsed in traumatic isolated full-thickness supraspinatus (SS) tears in young patients.

**Methods:**

One thousand twenty-six patients, who underwent arthroscopic shoulder surgery, were retrospectively reviewed. Pre-operative magnetic resonance imaging (MRI) of 69 patients aged 18 to 40 years with isolated traumatic full-thickness SS lesions remaining after exclusion criteria were evaluated for tendon retraction and atrophy grades. SS retraction was determined from a T2-weighted oblique coronal MRI slice, and the atrophy grade was determined from the T1-weighted oblique sagittal MRI slice. The patients were divided into four groups 0–1 month, 1–3 months, 3–6 months, and 6–12 months according to the time between trauma and MRI. The relationship of tendon retraction and muscle atrophy with elapsed time was evaluated, in addition, comparisons between groups were made.

**Results:**

Thirty-one (45%) of the patients were female and their mean age was 30 ± 7.3 (18–40) years. The mean age of men was 30.5 ± 6.9 (18–39) years (*p* = 0.880). The time between rupture and MRI was moderately correlated with retraction and strongly correlated with atrophy grades (*r* = 0.599, 0.751, respectively). It was observed that there was a statistically significant difference between the 1st (0–1 month) and 2nd (1–3 months) groups (*p* = 0.003, 0.001, respectively), and between the 2nd and 3rd (3–6 months) groups (*p* = 0.032, 0.002, respectively), but there was no significant difference between the 3rd and 4th (6–12 months) groups (*p* = 0.118, 0.057, respectively). In addition, there was a moderate correlation between tendon retraction and atrophy grades (*r* = 0.668). Power (1- b) in post hoc analysis was calculated as 0.826.

**Conclusions:**

The current study, supported by arthroscopy, showed that there is a moderate and strong positive correlation between the time elapsed after trauma and the level of retraction and degree of atrophy in traumatic full-thickness SS tears, and demonstrated the importance of early surgical intervention in young patients.

## Introduction

Rotator cuff tears (RCT) are among the shoulder pathologies that are frequently seen on admission to the clinic. [1] RCTs can be seen in all age groups and have the potential for disability if appropriate treatment is not given. [2] Although RCTs are seen between 9.4% and 39.0%, the incidence increases with aging. [3–7] Although the degenerative process is usually effective in RCT, the traumatic process is prominent in young patients. [8] Yamamoto et al. [9] showed a 5.1% incidence of RCT between the ages of 20 and 50 years. Milgrom et al. [10] reported that there were partial-thickness RCTs with an incidence of 8% between the ages of 30 and 50 with ultrasound evaluation. Similarly, Sher et al. [11] reported 4% of rotator cuff injuries under 40 years of age. In the same study, it was shown that asymptomatic tears are more common over the age of 40, but are very rare under the age of 40. RCTs in young and old patients’ healing potential, activity levels, etiology of the tear, and long-term prospects are different. [12–14]

Early surgical repair is recommended for young, active patients with acute and functional impairment. It is recommended that acute, traumatic, full-thickness RCT be treated before four months. (15–16) Thompson et al. [17] evaluated the relationship between tendon retraction and atrophy grade in their study comparing full-thickness supraspinatus (SS) tears with patients without tears. In this study, age and whether the tear was traumatic or not were not specified. In a similar study, Gilbert et al. [18] showed that fatty degeneration was positively correlated with tendon retraction in a study of full-thickness SS ruptures. The variation of retraction and atrophy over time in traumatic isolated full-thickness SS tears is unknown. No study was found examining the changes in atrophy and retraction levels elapsed time in isolated full-thickness SS tears in young patients. The main hypothesis in this study, planned in young patients for whom we know that early treatment is recommended for RCT, is that the timing of surgery in isolated SS tears can be determined according to the change in the degrees of retraction and atrophy over time. Since the progression of atrophy and retraction grades will negatively affect clinical results, it will be important to determine at what time intervals the treatment will be more effective. To prevent false negative or positive effects of MRI, planning was made for patients whose definitive diagnosis was made through shoulder arthroscopy by a single surgeon.

The current study aimed to determine the grade of retraction and atrophy according to the time elapsed in traumatic isolated full-thickness SS tears in young patients.

## Methods

**Fig. 1:**
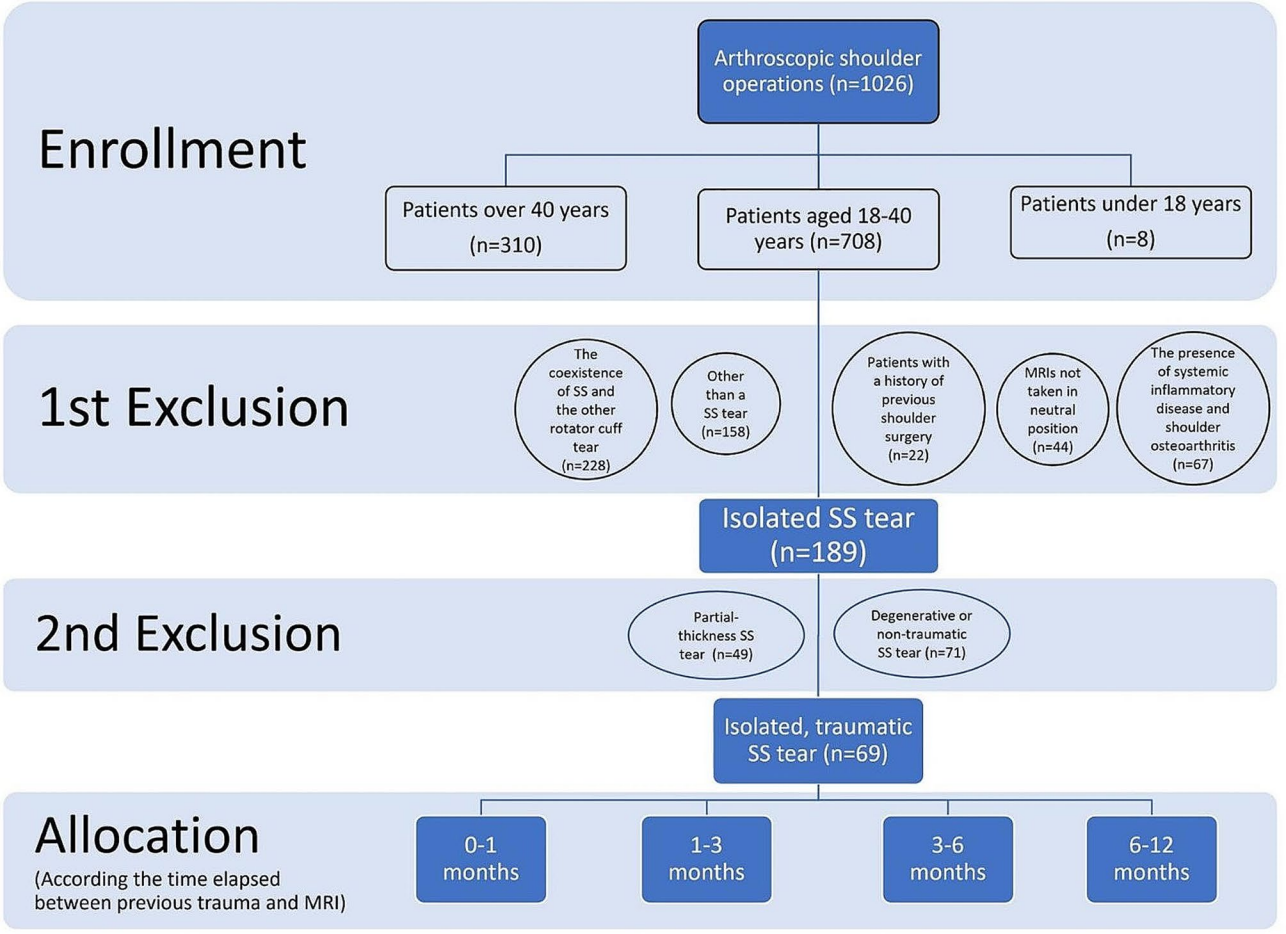
Flow-chart of cases

For this cross-sectional descriptive study, 1026 patients who underwent arthroscopic shoulder surgery by a single surgeon between 2015 and 2023 were retrospectively reviewed. To exclude additional subscapularis ruptures and degenerative SS tears that may have been missed in magnetic resonance imaging (MRI), only patients who underwent surgical treatment were included in the study. Patients aged between 18 and 40 years with traumatic isolated full-thickness SS tears were included in the study. The exclusion criteria were patients whose preoperative MRI scans could not be obtained and whose taken were not in a neutral position (*n* = 44), patients who were operated on for a reason other than a SS tear (*n* = 158), partial-thickness SS tear (*n* = 49), the coexistence of SS and the other rotator cuff tear (*n* = 228, 74 of these patients were found to have isolated SS tears on pre-operative MRI but were excluded due to additional rotator cuff ruptures during surgery) (94 association with subscapularis, 75 with infraspinatus, 52 with subscapularis and infraspinatus, 7 with subscapularis, infraspinatus, and teres minor), patients with degenerative or non-traumatic SS tear (*n* = 71), the presence of systemic inflammatory disease (*n* = 28), shoulder osteoarthritis (*n* = 39) [19], patients with a history of previous shoulder surgery (*n* = 22), patients over 40 years of age (*n* = 310), and being under 18 years of age (*n* = 8) (Fig. 1).

Preoperative MRIs of 69 (7%) patients with isolated traumatic full-thickness SS lesions were evaluated for tendon retraction and atrophy grades. Tendon retraction was measured as distance, and the grade of atrophy was calculated as a percentage. According to the anamnesis taken from the patients, it was learned that there were no complaints before the trauma and started after a certain trauma. The time elapsed between previous trauma and MRI was determined in patients with traumatic SS tears. According to this time, the patients were divided into four groups: 0–1 month, 1–3 months, 3–6 months, and 6–12 months. The 3–6 months group was further examined in three subgroups, such as 3–4 months, 4–5 months, and 5–6 months.

**Fig. 2:**
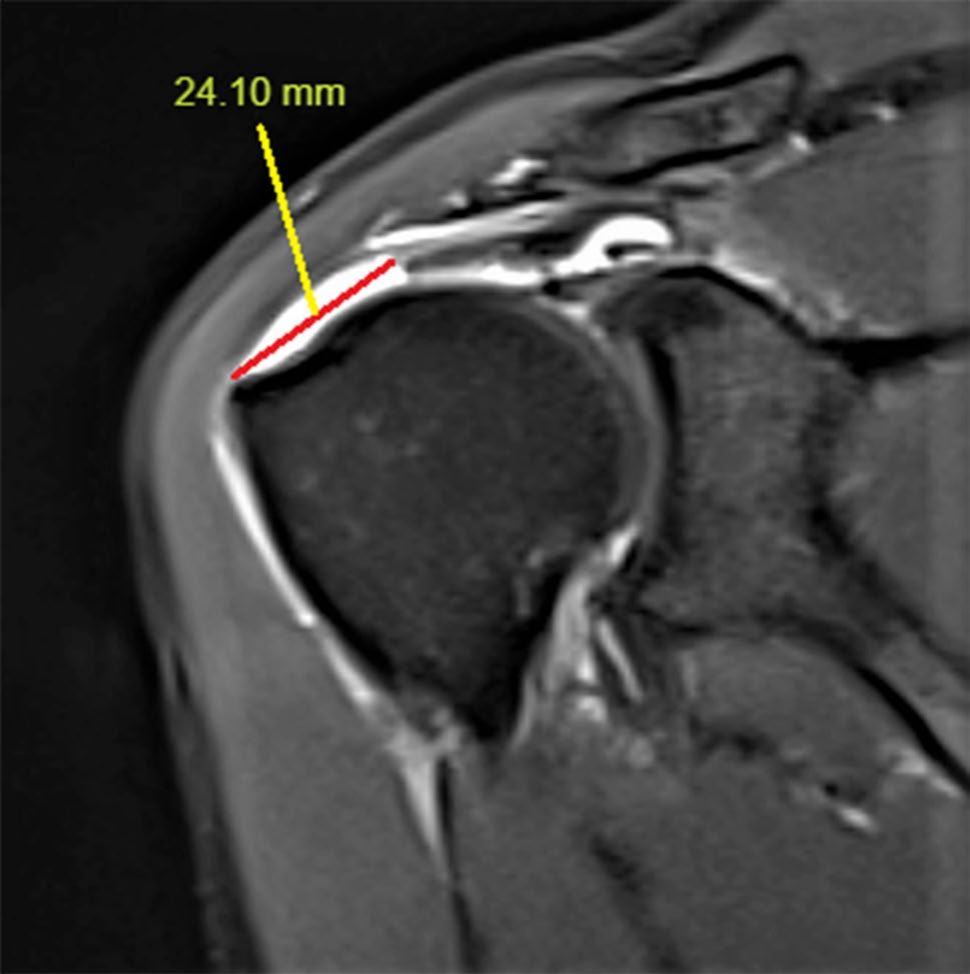
Supraspinatus tendon retraction measurement on a T2-weighted oblique coronal MRI slice. 24.1 mm retraction was measured in the given example

**Fig. 3:**
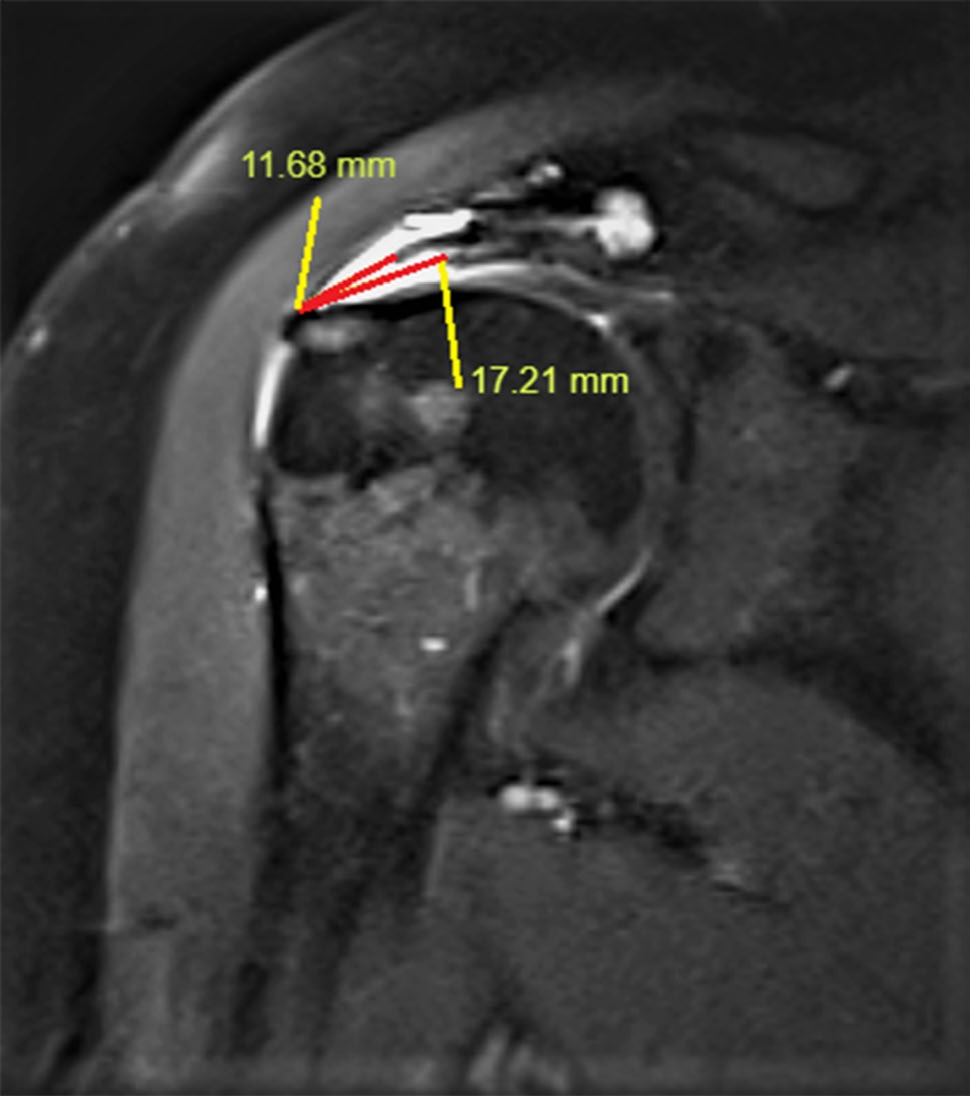
Two retraction levels of the delaminated supraspinatus tendon on T2-weighted oblique coronal MRI slice. 14.4 mm retraction was measured in the supraspinatus tear specimen with delamination (11.68/2 + 17.21/2)

SS retraction was determined from a T2-weighted oblique coronal MRI slice (Fig. 2), and the distance between the most lateral part of the tuberculum majus and the most lateral edge of the tendon was determined as SS retraction. Delamination can be defined as different levels of retraction of the bursal and articular surfaces of the rotator cuff tendons. There is no consensus on which layer to measure the retraction in case of delamination. Considering that both layers will affect atrophy, the middle of the two retraction levels was determined and included in the calculation (Fig. 3).

**Fig. 4:**
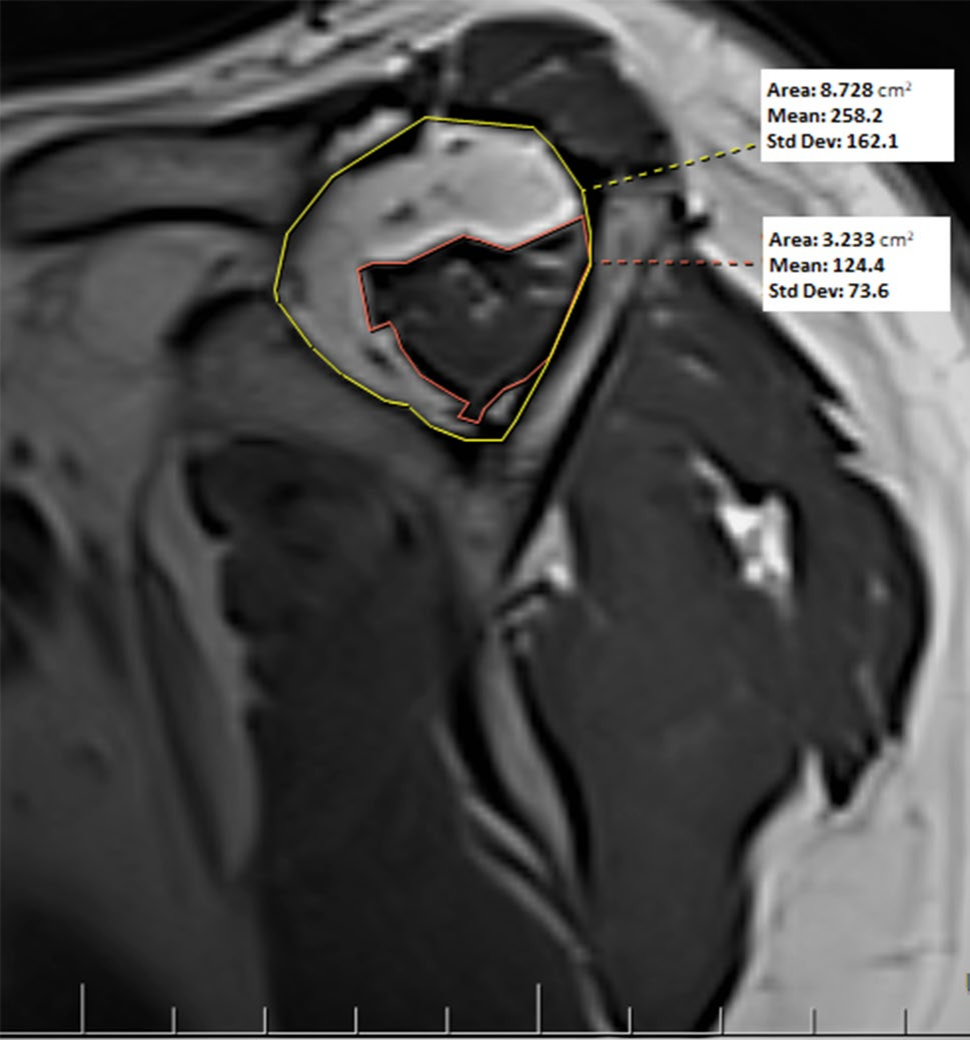
Supraspinatus muscle atrophy measurement on a T1-weighted oblique sagittal MRI slice. In the given patient sample, atrophy was calculated as 63% (8.73–3.23/8.73)

The atrophy grade was determined from the T1-weighted oblique sagittal MRI slice (Fig. 4). Fukuta et al. [20] stated that there may be a decrease in SS atrophy in more medial slices of the y-shape view in large tears. Therefore, in some cases, the measurement was made from this region since the part with the highest grade of atrophy is more medial to the Y-shaped view in the T1 oblique sagittal slice. In evaluating muscle atrophy, the measurement technique defined by Thomazeau et al. [21] was used, and the calculation was made as a ratio, not stages 1–3.

The relationship of tendon retraction and muscle atrophy with elapsed time was evaluated, in addition, comparisons between groups were made. The correlation between SS tendon retraction and atrophy was also added. In addition, age, and categorical data of the patients such as gender and which dominant hand was used, were noted. Acromion-type evaluation between groups was additionally performed. [22]

All MRI scans were done with a 1.5 Tesla unit (Siemens Magnetom Aera, Erlangen, Germany). T1-weighted and T2-weighted oblique coronal, T1-weighted and T2-weighted oblique sagittal, and T2-weighted axial images were obtained with 2 mm slice intervals. MRIs of all patients were performed using the same protocol and sequences. Shoulder MRI was performed in the neutral position in all patients. The neutral position was defined as having the arms at the sides of the body in the supine position with the thumb pointing up. Measurements were made on the existing Fonet Dicom Viewer v4.1 (Fonet Bilgi Teknolojileri A.Ş., Gölbaşı, Ankara, TR), and no additional software was used. All assessments were evaluated by two observers and inter-observer reliability was determined.

### Statistical analysis

SPSS (Statistical Package for the Social Sciences) version 24 (IBM Corp., Armonk, New York, USA) was used for statistical analysis. Shapiro Wilk test was applied to the measurements to be evaluated for normality analysis. Mann Whitney U analysis was used for non-normally distributed parameters, and an independent t-test was used for those with normal distribution, a p-value less than 0.05 was considered significant. The Pearson Coefficient was used to determine the correlation, an r-value below 0.3 was considered very weak, between 0.3 and 0.5 weak, 0.5–0.7 moderate, and above 0.7 strong correlation. [23] The Fleiss kappa (k) coefficient was used for the inter-observer reliability. A k value is always between 0 and 1; the higher k value indicates a better correlation. The k values were graded as slight (0-0.2), fair (0.21–0.40), moderate (0.41–0.60), substantial (0.61–0.80), and almost perfect (0.81-1). Post hoc analysis was performed using the G*power 3.1.9.7 program (Heinrich-Heine-Universität Düsseldorf, GERMANY).

## Results

### Demographic data of the patients

Thirty-one (45%) of the patients were female and their mean age was 30 ± 7.3 (18–40) years. The mean age of men was 30.5 ± 6.9 (18–39) years (*p* = 0.880). Forty-nine (71%) of the injuries were in the dominant arm. Considering the time between rupture and MRI, nine patients were in 1st group (0–1 month), 15 patients in 2nd (1–3 months), 24 patients were in the 3rd (3–6 months), and 21 patients were in the 4th group (6–12 months). It was seen that 23 patients had type 1 acromion, 33 patients had type 2, and 13 patients had type 3 (Table 1).

**Table 1:** Demographic data of patients grouped by the time between rupture and magnetic resonance imaging

	1st group (0–1 mh)	2nd group (1–3 mh)	3rd group (3–6 mh)	4th group (6–12 mh)	Total
Age mean ± SD (range)	26.1 ± 7 (18–37)	33.2 ± 7.3 (19–39)	32.3 ± 6.6 (19–38)	27.5 ± 5.9 (18–40)	30.2 ± 7 (18–40)
Sex female/male (n)	5/4	6/9	9/15	11/10	31/38
Dominant arm/non-dominant arm (n)	5/4	9/6	16/8	19/2	49/20
Acromion type (1/2/3)	5/3/1	4/6/5	6/13/5	7/11/2	23/33/13
Total (n)	9	15	24	21	69

SD: standard deviation, mh: month

### Evaluation of main parameters

When the atrophy percentages were evaluated, it was seen that the 0–1-month group was 12.6 ± 4.88, the 1–3-month group was 24.87 ± 8.53, the 3–6-month group was 35.58 ± 10.2, and the 6–12-month group was 40.38 ± 5.07. When retraction levels were evaluated, it was seen that it was 7.46 ± 1.86 in the 0–1-month group, 12.92 ± 4.59 in the 1–3-month group, 16.7 ± 5.49 in the 3–6-month group and 19.5 ± 6.28 in the 6–12-month group.

The time between rupture and MRI was moderately correlated with retraction and strongly correlated with atrophy grades (*r* = 0.599, 0.751, respectively) (Table 2). In addition, the retraction and atrophy grades of the rupture groups were compared among themselves. It was observed that there was a statistically significant difference between the 1st and 2nd groups (*p* = 0.003, 0.001, respectively), and between the 2nd and 3rd groups (*p* = 0.032, 0.002, respectively), but there was no significant difference between the 3rd and 4th groups (*p* = 0.118, 0.057, respectively) (Table 3). While there was a statistically significant difference between the 2nd and 3rd groups, the 3rd group was evaluated separately by grouping it within itself, since the significant difference between the 3rd and 4th groups disappeared. When the 3rd group (3–6 months) was divided into three groups (A: 3–4 months, B: 4–5 months, and C: 5–6 months), there was a statistically significant difference between the atrophy grades of the A and B groups (*p* = 0.015), while no statistical difference was found in the retraction levels (*p* = 0.082). No statistical differences were found in groups B and C (*p* = 0.481 retraction, *p* = 0.507 atrophy) (Table 4).

**Table 2:** The correlation of parameters with retraction and atrophy grades

	*r* value			
	The retraction level	Interpr.	The atrophy grade	Interpr.
The rupture groups	0.599	Moderate corr.	0.751	Strong corr.
Age	− 0.180	Very weak corr.	− 0.153	Very weak corr.
Sex	0.098	Very weak corr.	− 0.036	Very weak corr.
The acromion type	0.127	Very weak corr.	− 0.010	Very weak corr.

Interpr: Interpretation, corr: correlation

**Table 3:** Comparison of retraction and atrophy grades of rupture groups among themselves

	*P* value	
	The retraction level	The atrophy level
1st – 2nd groups	0.003	0.001
1st – 3rd groups	< 0.001	< 0.001
1st – 4th groups	< 0.001	< 0.001
2nd − 3rd groups	0.032	0.002
2nd – 4th groups	0.002	< 0.001
3rd – 4th groups	**0.118**	**0.057**

*1st :0–1 month*,* 2nd :1–3 months*,* 3rd : 3–6 months*,* 4th : 6–12 months*

**Table 4:** Comparison of retraction and atrophy grades of the 3rd group (3–6 months) by months

	A	B	C	*p*-value (A-B)	*p*-value (B-C)	*p*-value (A-C)
Retraction (mm) mean ± SD	12.65 ± 2.88	17.6 ± 6.8	19.6 ± 4.24	0.082	0.481	**0.001**
Atrophy (%) mean ± SD	26.75 ± 4.1	38.14 ± 10.71	41.44 ± 8.69	**0.015**	0.507	**0.001**
n	8	7	9			

*A: 3–4 months*,* B: 4–5 months*,* C: 5–6 months*,* SD: standard deviation*

### Evaluation of other parameters and post hoc analysis

There was a very weak negative correlation between age and retraction and atrophy grades (*r*=-0.180, − 0.153, respectively). Similarly, there was a very weak correlation between sex and retraction and atrophy grades (*r* = 0.098, − 0.036, respectively) (Table 2).

Similarly, there was a very weak negative correlation between acromion types and rupture groups (*r*=-0.015). There was a very weak correlation between acromion type and retraction (*r* = 0.127), and a very weak negative correlation between atrophy grades (*r*=-0.010) (Table 2).

There was a moderate correlation between tendon retraction and atrophy grades (*r* = 0.668).

The retraction and atrophy grades were found to be in substantial agreement in the inter-observer evaluation (κ = 0.785, 0.624, respectively).

Power (1- b) in post hoc analysis was calculated as **0.826** (*n* = 69, effect size = 0.3, a err prob = 0.05).

## Discussion

It is known that young-age RCTs are more likely to occur on the basis of trauma. [8] In this current study, the changes in retraction and atrophy degrees over time in young patients with traumatic full-thickness SS tears were examined, it was determined that there was a moderate positive correlation between the elapsed time with the retraction level, and a strong positive correlation with atrophy grades (*r* = 0.599, *r* = 0.751, respectively). Additionally, it has been determined that the 4th month is a critical threshold for atrophy level.

Thompson et al. [17] compared the MR views of 143 patients without a tear in the SS tendon and 148 patients with tendon retraction accompanying a full-thickness tear. It was determined that there was a statistically significant increase in the Goutallier grade in the rupture group (*p* < 0.001) and the increase in the Goutallier grade was associated with retraction (*p* < 0.001). In the same study, it was observed that retraction increased significantly if the Goutallier grade increased from three to four (19.5–34.7 mm). When the relationship between the 3–4 months and 4–5 months groups was examined in the current study, a statistically significant increase in the grade of atrophy was detected (*p* = 0.015), while the difference between the retraction levels was found to be insignificant (*p* = 0.082). However, when all periods were evaluated, a moderate positive correlation was found between the increasing grade of atrophy and tendon retraction (*r* = 0.668).

A systematic review stating that the definition of “acute and/or traumatic RCT” is contradictory in the literature, is aimed to provide a standard definition for these expressions. [24] The 18 articles reviewed in this review were divided into two groups, 10 studies using a minimum duration of two to a maximum of six weeks for acute RCTs, and eight studies using a duration of two to six months. In a randomized controlled trial with level-1 evidence cited in the same review, Ranebo et al. [25] used the 3-month limit for 58 patients. In the current study, we divided the traumatic full-thickness isolated SS rupture patients into four different time groups in order not to use the contradictory “acute” expression with precise time intervals.

Petersen et al. [15] in their study examining the functions of acute traumatic full-thickness RCTs, it was reported that fat atrophy and tear size were not associated with postoperative function if repaired before four months. Similarly, Patel et al. [16] reported that repairing acute traumatic full-thickness RCTs within four months of injury gave better results. In the current study, there was a statistically significant difference between the 2nd group (1–3 months) and the 3rd group (3–6 months) (*p* = 0.032 [retraction], *p* = 0.002 [atrophy]), while the absence of a significant difference between the 3rd group and the 4th group (6–12 months) (*p* = 0.118 [retraction], *p* = 0.057 [atrophy]) supports this situation. In addition, when the 3rd group (3–6 months) was divided into 3 groups and compared, a statistically significant increase was found in atrophy grades when it was passed from 3 to 4 months to 4–5 months (*p* = 0.015).

Paul et al. [26] in a study comparing traumatic and non-traumatic RCTs, stated that traumatic tears are more common in younger patients and show less muscle atrophy, fat degeneration, and tendon retraction, although they are seen in larger tear sizes. Paul et al. examined 28 traumatic RCTs, consisting of 28 supraspinatus, 11 infraspinatus, and four subscapularis tears, with a mean age of 43.8 years. In the current study, which examined 69 patients with isolated SS traumatic tears, with a mean age of 30.2, it was observed that retraction and atrophy were correlated with time in young patients. Time after trauma was highly correlated with the grade of retraction and atrophy (*r* = 0.599 [retraction], *r* = 0.751 [atrophy]), but no comparison was made with non-traumatic ruptures.

Gilbert et al. [18] in a study, they conducted with full-thickness SS ruptures, examined the relationship of fatty degeneration with tendon thickness and retraction. They stated that fatty degeneration showed a positive correlation with tendon retraction and a negative correlation with tendon thickness. In the current study, there was a moderate correlation between tendon retraction and atrophy grades (*r* = 0.668).

Bierry et al. [27] investigated the retraction pattern in non-delaminated and delaminated full-thickness RCTs in a study they conducted. According to the results of the study, it was observed that the retraction in the non-delamination group (31.5 mm) was significantly less than the articular surface retraction (36.3 mm) and higher than the bursal surface retraction (21 mm) of the delamination group (*p* < 0.0001). In the current study, the middle of two retraction levels was determined in patients with delamination and included in the calculations.

This study has some limitations. A limited number of patients have been evaluated. The mechanism of trauma is not specified, which may influence retraction. Since MRI scans are performed at 2 mm intervals as standard, better calculations can be made with thinner slices. Due to the retrospective study, these deficiencies can be corrected with a prospective design. To exclude additional subscapularis ruptures and degenerative SS tears that might be missed on MRI, the evaluation of patients who underwent surgical treatment alone was considered the strongest aspect of the study.

## Conclusion

The current study, supported by arthroscopy, showed that there is a moderate and strong positive correlation between the time elapsed after trauma and the level of retraction and degree of atrophy in traumatic full-thickness SS tears, and demonstrated the importance of early surgical intervention in young patients. It was also determined that the 4th month was a potential threshold.

## Data Availability

The corresponding author can provide data when necessary.

## Abbreviations

SS: Supraspinatus
RCT: Rotator Cuff Tears
MRI: Magnetic Resonance Imaging
